# Oncology provider experiences during the COVID-19 pandemic

**DOI:** 10.1371/journal.pone.0270651

**Published:** 2022-07-26

**Authors:** Hannah Arem, Jenna Moses, Larissa Nekhlyudov, Maureen Killackey, Beth Sieloff, Cindy Cisneros, Mandi L. Pratt-Chapman

**Affiliations:** 1 Healthcare Delivery Research, Medstar Health Research Institute, Washington, DC, United States of America; 2 Department of Epidemiology, George Washington University Milken Institute School of Public Health, Washington, DC, United States of America; 3 George Washington University Cancer Center, Community Advisory Board, Washington, DC, United States of America; 4 Department of Medicine, Brigham and Women’s Hospital, Harvard Medical School, Boston, MA, United States of America; 5 American College of Surgeons’ Commission on Cancer Site Reviewer, NYS Cancer Advisory Council, NY, United States of America; 6 Inter-Tribal Council of Michigan, Sault Ste, MI, United States of America; 7 School of Medicine and Health Sciences, George Washington University, Washington, DC, United States of America; Bay Area Hospital, North Bend Medical Center, UNITED STATES

## Abstract

**Purpose:**

The COVID-19 pandemic upended nearly all aspects of daily life and of medical care, placing a double burden of professional and personal concerns on those who provide medical care. We set out to assess the burden of the pandemic on provider outlook and understand how cancer survivorship providers experienced rapid changes to practice.

**Methods:**

We distributed a survey through the American College of Surgeons Commission on Cancer (CoC) to its accredited organizations in mid-October 2020. We included questions on provider characteristics, changes in patient care practices resulting from the pandemic, worry about COVID-19, and concern about impact on cancer survivors.

**Results:**

Of the n = 607 participants, three-quarters were female and three-quarters were White. Only 2.1% of participants reported having had COVID-19, but 43% reported anxiety about getting COVID-19 and over a quarter experienced sadness or depression, anxiety about the future, changes to sleep, difficulty concentrating, or social isolation. Approximately half of providers also expressed significant concern about progression of cancer in patients who experienced care delays or were afraid of accessing in-person care. In terms of changes to survivorship care, respondents reported changes to visitor policies, delays or cancellations, and efforts to reduce in-person visits.

**Conclusions:**

COVID-19 has taken a significant toll on front-line healthcare professionals, including oncologists and cancer care allied health professionals. Findings support proactive mental health support of healthcare professionals as well as emergency preparedness to manage delays to care for cancer patients in the event of future unexpected pandemics.

## Introduction

The COVID-19 pandemic upended nearly all aspects of daily life, including medical care for cancer survivors. Immediate and widely implemented changes included rapid shifts to telehealth, delays in screenings and treatment, and changes in visitor policies [1, 2]. Rapid publications, including from professional societies, detailed how to adapt oncology care, with recommendations for change in delivery of those services that could be delivered remotely, as well as services that were not time sensitive (e.g. delaying adjuvant therapy), vs those that should not be delayed (e.g. curative treatment for solid tumors) [1, 3–8]. Editorials outlined opportunities for telemedicine in future oncology care, as well as how to resume and improve cancer survivorship care to address needs unmet during the pandemic [3, 9, 10].

However, there are few studies published on cancer care provider experiences during the pandemic. Of the relevant publications, two focused on providers for childhood cancer patients [11, 12], another focused on breast cancer physicians only [13], while another study focused on caring specifically for older adults with cancer [14]. Our study builds on this emerging body of literature to describe experiences of a nationwide sample of oncology providers (physicians, PAs, nurses, social workers, etc.) caring for adult-onset cancer survivors, including delays to care and the extensive personal toll of providing care during the pandemic. Ours is the first known study to examine provider perspectives on care during the COVID-19 pandemic focused on the post-treatment cancer survivorship phase.

## Methods

### Survey development

The Cancer Care Provider Experiences with COVID-19 Survey was developed by the authors (HA and MPC) through a mix of consensus-based, adapted and validated measures (S1 Table). Two screening questions verified eligibility (i.e., active provider of cancer survivorship care and practice within the U.S.). The 43-item survey included 11 demographic questions and skip-logic for outcome variables to reduce length of the survey for respondents based on individual experiences. Questions asked about provider characteristics, changes in patient care practices resulting from the pandemic, worry about COVID-19 including mental health concerns (adapted from National Institutes of Health PhenX toolkit on COVID-19 questionnaire) [15], and concerns about the impact of COVID-19 on patient outcomes.

### Recruitment

The survey was distributed through the American College of Surgeons Commission on Cancer (CoC) to its accredited organizations. The CoC sent the survey to the cancer registrar and physician liaison on record at each cancer center and asked the registrar to forward the survey to the survivorship coordinator. Using the Dillman method, the survey was disseminated via email three times from October 13 to October 27, 2020, one week apart [16, 17]. Each email included a direct link to the survey in the Research Electronic Data Capture (REDCap) system. Study data were collected and managed using REDCap electronic data capture tools hosted at Children’s National [18]. REDCap is a secure, web-based application designed to support data capture for research studies, providing 1) an intuitive interface for validated data entry; 2) audit trails for tracking data manipulation and export procedures; 3) automated export procedures for seamless data downloads to common statistical packages; and 4) procedures for importing data from external sources.

### Study population

There were 966 observations in the provider data. First, we eliminated n = 145 who did not report practicing in the US, and second, we eliminated n = 13 who did not report providing care for post-treatment cancer survivors specifically. We then eliminated individuals who had large amounts of missing data, which we defined as those who answered no questions (i.e. opened the survey but did not complete any fields, or those who completed only the first question, but no further questions out of the 11 questions that were focused on changes due to the pandemic), which left us with a final dataset of N = 607.

### Statistical analyses

We used descriptive statistics (means, percentages) to describe responses among our sample population. SAS V9.4 (Cary, NC) was used in all analyses.

The study received IRB exemption from the George Washington University (NCR202819). All participants had to acknowledge reviewing and agreeing to the informed consent presented on the survey landing page in REDCap via a checkbox before proceeding with the survey. Funding for the study was provided by the Patient Centered Outcomes Research Institute (PCORI EADI-12744).

## Results

Respondent characteristics are described in Table 1. Of the N = 607 respondents, 24.1% self-reported male and 59.3% self-reported female gender. Most respondents were non-Hispanic White (75.6%). Respondents were relatively evenly distributed by urban/suburban status, with 39.9% reporting urban locations, 35.8% reporting suburban locations, with a lower percentage (18.0%) reporting rural locations. Providers came from varied perspectives, including medical oncology (8.6%), surgical oncology (13.5%), radiation oncology (4.1%), nursing (25.5%), nurse practitioner/physician’s assistant (NP/PA) (19.4%), social work (6.1%), and patient navigation (13.3%). Most respondents were associated with a COC-accredited organization (78.8%), while some reported association with a community hospital (44.2%) and some with an academic or teaching hospital (18.3%) (not mutually exclusive).

**Table 1:** Characteristics of survey participants (N = 607)^a^.

**Provider sex assigned at birth**	
Male	113 (18.6)
Female	447 (73.6)
Prefer not to answer	8 (1.3)
Missing	39 (6.4)
**Gender**	
Cisgender Male	150 (24.1)
Cisgender Female	360 (59.3)
Other	5 (0.9)
Prefer not to answer	28 (4.6)
Do not understand the question	25 (4.1)
Missing	39 (6.4)
**Sexual Orientation**	
Lesbian, Gay, Homosexual, Bisexual, Pansexual	11 (1.8)
Straight	524 (86.3)
Prefer not to answer	32 (5.3)
Do not understand the question	3 (0.5)
Missing	37 (6.1)
**Race/ethnicity**	
Asian	36 (5.9)
Black	15 (2.5)
Hispanic/Latinx	38 (6.3)
White	459 (75.6)
Other	12 (2.0)
I prefer not to answer	22 (3.6)
Missing	25 (4.1)
**Urbanicity**	
Urban (city)	242 (39.9)
Suburban (outside a city)	217 (35.8)
Rural (not near a city)	109 (18.0)
Missing	39 (6.4)
**Location**	
State	551 (90.8)
Tribe/Territory	15 (2.5)
Missing	41 (6.7)
**Role**	
Oncologist	159 (26.2)
Other physician	22 (3.6)
Nurse practitioner/ Physician assistant	118 (19.4)
Nurse	155 (25.5)
Clinical Researcher	7 (1.2)
Social Worker	37 (6.1)
Patient Navigator	81 (13.3)
Missing	28 (4.6)
**Organization** ^**a**^	
CoC-accredited	478 (78.8)
NCI-designated	50 (8.2)
Academic/Teaching hospital	111 (18.3)
Community hospital	268 (44.2)
Free-standing oncology practice	46 (7.6)
Free-standing multi-specialty group	17 (2.8)
Other cancer practice	20 (3.3)

^a^Participants can select more than one category.

Providers reported a number of personal concerns during the pandemic (Table 2). The most common concerns were anxiety about getting COVID-19 (42.8%; range 50.3% for physicians to 36.6% for NP/PAs), infecting others (43.0%), and concern about a family member or close friend getting or dying of COVID-19 (53.1%). Still, most providers also reported hopefulness that the pandemic would end soon (53.2%). Notably, only 34.3% (range 43.6% for physicians to 26.6% for nurses) reported believing that there will be a vaccine or cure for COVID soon, although FDA approval for the Pfizer-BioNTech COVID-19 vaccine would prove to be a little more than a month away from the time of survey completion. Approximately a quarter to a third of providers reported feelings of sadness or depression, negativity or anxiety about the future, changes to sleep, changes in eating habits or weight, concentration issues, and social isolation. Still 57% reported engaging in mindfulness activities and/or a hobby that brought joy to their life (range 59.8% for physicians to 50.9% for NP/PAs).

**Table 2:** Personal concerns during the pandemic, overall and by provider role^a^.

	Overall	Physician	NP/PA	Nurse	Other^b^
Anxiety/Worry related to COVID-19 (scale 0–11), mean, sd	3.4 (2.7)	3.5 (2.7)	3.2 (2.9)	3.7 (2.7)	3.5 (2.7)
	**N (%)**				
I feel anxious about getting COVID-19	260 (42.8)	90 (50.3)	41 (36.6)	47 (43.1)	53 (46.1)
I have been diagnosed with COVID-19	13 (2.1)	8 (4.5)	3 (2.7)	2 (1.8)	0 (0)
I worry about the possibility of infecting others	261 (43.0)	84 (46.9)	45 (40.2)	57 (52.3)	46 (40.0)
I am concerned about a family member or close friend getting or dying from COVID-19	322 (53.1)	103 (57.5)	51 (45.5)	66 (60.6)	55 (47.8)
A family member or close friend has died from COVID-19	53 (8.7)	14 (7.8)	9 (8.0)	10 (9.2)	10 (8.7)
I worry about the possibility of dying from COVID-19	90 (14.8)	32 (17.9)	14 (12.5)	13 (11.9)	17 (14.8)
I feel I have no control over how COVID-19 will impact my life	130 (21.4)	30 (16.8)	28 (25.0)	21 (19.3)	33 (28.7)
I believe there will be a vaccine or cure for COVID-19 soon	208 (34.3)	78 (43.6)	34 (30.4)	29 (26.6)	33 (28.7)
I am hopeful that the COVID-19 pandemic will end soon	323 (53.2)	85 (47.5)	60 (53.6)	53 (48.6)	66 (57.4)
None of these apply to me	33 (5.4)	8 (4.5)	7 (6.3)	6 (6.4)	6 (5.2)
I have experienced feelings of sadness or depression	167 (27.5)	48 (26.8)	28 (25.0)	37 (33.9)	30 (26.1)
I feel negative and/or anxious about the future	189 (31.1)	58) 32.4)	38 (33.9)	29 (26.6)	39 (33.9)
I have experienced changes in my sleep	170 (28.0)	52 (29.1)	27 (24.1)	34 (31.2)	31 (27.0)
I have experienced changes in my eating and/or weight changes	140 (23.1)	39 (21.8)	29 (25.9)	28 (25.7)	28 (24.4)
I have experienced difficulty concentrating	154 (25.4)	38 (21.2)	25 (22.3)	35 (32.1)	35 (30.4)
I have experienced feelings of social isolation or loneliness	182 (30.0)	61 (34.1)	27 (24.1)	32 (29.4)	33 (28.7)
I am hopeful about the future	336 (55.4)	106 (59.2)	62 (55.4)	60 (55.1)	59 (51.3)
I make an effort to engage in mindfulness activities like meditation and/or yoga	237 (39.0)	70 (39.1)	44 (39.3)	37 (33.9)	51 (44.4)
I have a hobby that brings joy to my life	346 (57.0)	107 (59.8)	57 (50.9)	63 (57.8)	67 (58.3)
None of these apply to me	35 (5.8)	8 (4.5)	9 (8.0)	7 (6.4)	6 (5.2)

^a^Participants were instructed to check all that apply

^b^Other: Clinical researcher, social worker, patient navigator.

When asked about long-term concerns of the pandemic (Table 3), 14.2% of providers were very worried and 49.3% of provider were somewhat worried (range 19.8% of physicians vs 10.6% of nurses), while 31.3% were not worried about the healthcare system handling backlogged patients (range 38.5% of nurse vs 29.3% of NP/PAs). A greater percentage were very (46.8%; range 60% of other providers vs 52.9% of nurses) or somewhat (38.7%) worried about progression of cancer in patients who experienced care delays; these numbers were similar to worry about progression of cancer in patients afraid of accessing care.

**Table 3:** Long-term effects of the pandemic [to be published online only].

How concerned are you about the following issues after physical distancing protocols are lifted?					
	Overall	Physician	NP/PA	Nurse	Other
Inability for system to handle backlogged patient needs					
*Very*	86 (14.2)	34 (19.8)	17 (16.0)	11 (10.6)	13 (12.0)
*Somewhat*	299 (49.3)	81 (47.1)	58 (54.7)	53 (51.0)	61 (56.5)
*Not*	190 (31.3)	57 (33.1)	31 (29.3)	40 (38.5)	34 (31.5)
Progression of cancer in patients who experienced care delays					
*Very*	284 (46.8)	72 (42.1)	54 (50.9)	51 (48.6)	56 (51.9)
*Somewhat*	235 (38.7)	83 (48.5)	40 (37.7)	46 (43.8)	39 (36.1)
*Not*	55 (9.1)	16 (9.4)	12 (11.3)	8 (7.6)	13 (12.0)
Progression of cancer in patients afraid of accessing in-person care					
*Very*	324 (53.4)	92 (53.5)	63 (58.9)	55 (52.9)	63 (60.0)
*Somewhat*	220 (36.2)	70 (40.7)	39 (36.5)	48 (46.2)	36 (34.3)
*Not*	29 (4.8)	10 (5.8)	5 (4.7)	1 (1.0)	6 (5.7)

Providers reported significant changes in patient care during the pandemic (Table 4). Nearly all reported changes to visitor policies (93.6%), while many reported delays in follow up for patients in treatment (64.6%) or survivorship care (66.4%). Approximately half reported changes to treatment selection to reduce in-person visits (47.0%) and reported triage based on professional society guidelines (e.g. NCCN, ACOS, ASCO) to determine when in-person visits were needed (53.2%). One quarter of respondents reported suspension of non-urgent clinical trials (26.4%) and a third reported team-based staff schedules to avoid potential cross-team COVID-19 exposures (33.1%).

**Table 4:** Reported practice changes due to COVID, overall and as reported by provider role^a^.

	Overall	Physician	NP/PA	Nurse	Other
	n (%)	n (%)	n (%)	n (%)	n (%)
Follow up for patients in treatment delayed or cancelled	392 (64.6)	129 (72.1)	68 (60.7)	69 (63.3)	70 (60.9)
Post-treatment survivorship care canceled or delayed	403 (66.4)	117 (65.4)	84 (75.0)	65 (59.6)	77 (67.0)
Remote work for non-clinical staff	451 (74.3)	137 (76.5)	74 (66.1)	83 (76.2)	74 (80.4)
Triage based on specialty guidelines (e.g. NCCN, ACOS, ASCO) to ensure clinical need for in-person visits	323 (53.2)	117 (65.4)	66 (58.9)	49 (45.0)	45 (39.1)
Changes to treatment selection to reduce in-person visits	285 (47.0)	114 (63.7)	42 (37.5)	35 (32.1)	49 (42.6)
Team-based staff schedules to avoid potential cross-team exposure to COVID-19	201 (33.1)	71 (40.0)	36 (32.1)	27 (24.8)	35 (30.4)
Suspension of nonurgent clinical trials	160 (26.4)	68 (38.0)	27 (24.1)	20 (18.4)	17 (14.8)
Changes to visitor policies (e.g. restricting appointment companions)	568 (93.6)	167 (93.3)	101 (90.2)	106 (97.3)	112 (97.4)
Other	46 (7.6)	8 (4.5)	7 (6.3)	8 (7.3)	9 (7.8)

^a^Participants were instructed to check all that apply.

## Discussion

We found that the COVID-19 pandemic affected cancer survivorship care practice and provider emotional and practical experiences. We identified ongoing concerns as states remain under restrictions and as the U.S. begins to emerge from the pandemic. In terms of practice changes, similar to our findings, other studies have also reported delays to cancer treatment for older adults [14, 19] and appointments for cardio-oncology [20], as well as for treatment of hematological malignancies in one middle-income country [21].

Our study further supports a growing compilation of findings on the personal and professional toll of the pandemic on a broad spectrum of oncology providers. Even prior to the pandemic there were concerns about burnout among oncology health professionals [22–26]. There have further been a number of opinion papers about the impact of COVID-19 on mental health [27, 28], and particularly the burden and stresses placed on healthcare providers, including oncologists [13, 29–34]. An early study out of China suggested symptoms of depression, anxiety, insomnia and distress in health care workers exposed to COVID-19 in March of 2020 [35]. In addition, a study of radiation oncologists in India [36] and of medical oncologists in Canada [37] corroborate our findings that providers worried more about exposing loved ones to COVID-19 than they did about their own personal exposure to COVID. A New Jersey-based study of childhood oncology providers and staff also reported high burnout, but also the significance of pre-pandemic burnout [12]. Interestingly, this study reported that 87% of participants reported that mental health resources were available but only 8.4% had utilized them [12]. Our study indicated less personal concern of contracting COVID than a study conducted in Canada in the early phase of the pandemic (April 2020), but similar rates of reported COVID-19 acquisition. In our study over a quarter of respondents experienced mental health concerns similar to those reported by providers during other infectious disease outbreaks (e.g. severe acute respiratory syndrome- SARS) such as sadness or depression, negativity or anxiety about the future, difficulty concentrating, and social isolation [38]. Given that even prior to the pandemic, physicians were subject to greater burnout relative to the general population, supporting health care provider work-life balance is a critical need [39]. Recent publications highlight strategies for implementing institutional well-being programs during the COVID-19 pandemic and in the future [40], but implementation research is needed to better determine how to introduce these strategies and how they might reach the whole oncology team [33]. Our study also demonstrated differences in perceived worry or anxiety by provider role. Further studies are needed to understand the longer-term impact of these mental health concerns and to implement programs to support providers with lingering mental health impacts from this pandemic or to prepare to support providers through future pandemics, accounting for how different roles may be differently impacted.

A recently published consensus piece focused on cancer surgery from the Internal Cancer Benchmarking Partnership warned of pending needs and heightened caseloads due to COVID-related delays, specifically recommending prompt screening procedures and timely surgeries as needed, reducing diagnosis to treatment intervals [3]. In our survey, similarly, an overwhelming majority of providers were concerned about progression of cancer in patients who experienced care delays and those afraid of accessing in-person care.

Strengths of our study include the large nationwide sample of a diverse group of cancer care providers (e.g. oncologists, NPs, patient navigators), and data captured approximately 6 months into the COVID-19 worldwide pandemic. Limitations of our study include the single time point of assessment, as well as the need to adapt validated scales given limitations in the existing scales to measure the impact of the pandemic on cancer provider experiences.

## Conclusion

Our survey results confirm findings that provider burnout and burden are ongoing concerns in the health profession, including oncologists and cancer care allied health professionals, and that the COVID-19 pandemic placed an additional burden on these individuals. This study is among the first to report changes to practice and mental health concerns resulting from COVID-19 among a national sample of oncology care providers and the first to report on the experiences of cancer survivorship care practitioners, specifically. Findings support proactive mental health support of healthcare professionals who are on the front line of pandemic responses as well as emergency preparedness to manage delays to care for cancer patients in the event of future unexpected pandemics.

## Supporting information

S1 Table Provider survey. (PDF)

## References

[1] Schrag D, Hershman DL, Basch E. Oncology Practice During the COVID-19 Pandemic. JAMA. 2020;323(20):2005–6. doi: 10.1001/jama.2020.6236 32282023

[2] Brugel M, Carlier C, Essner C, Debreuve-Theresette A, Beck M-F, Merrouche Y, et al. Dramatic Changes in Oncology Care Pathways During the COVID-19 Pandemic: The French ONCOCARE-COV Study. The Oncologist. 2021;26(2):e338–e41. doi: 10.1002/onco.13578 33111460 PMC7873337

[3] Butler J, Finley C, Norell CH, Harrison S, Bryant H, Achiam MP, et al. New approaches to cancer care in a COVID-19 world. Lancet Oncol. 2020;21(7):e339–e40. doi: 10.1016/S1470-2045(20)30340-5 . 32615112 PMC7324090

[4] Ueda M, Martins R, Hendrie PC, McDonnell T, Crews JR, Wong TL, et al. Managing cancer care during the COVID-19 pandemic: agility and collaboration toward a common goal. Journal of the National Comprehensive Cancer Network. 2020;18(4):366–9. 10.6004/jnccn.2020.7560 32197238

[5] Chan A, Ashbury F, Fitch MI, Koczwara B, Chan RJ, Group MSS. Cancer survivorship care during COVID-19—perspectives and recommendations from the MASCC survivorship study group. Support Care Cancer. 2020:1–4. PubMed Central PMCID: PMC7247777. doi: 10.1007/s00520-020-05544-4 32451702 PMC7247777

[6] Pennell NA, Dillmon M, Levit LA, Moushey EA, Alva AS, Blau S, et al. American Society of Clinical Oncology Road to Recovery Report: Learning From the COVID-19 Experience to Improve Clinical Research and Cancer Care. Journal of Clinical Oncology. 2021;39(2):155–69. doi: 10.1200/JCO.20.02953 . 33290128 PMC12451922

[7] Riera R, Bagattini ÂM, Pacheco RL, Pachito DV, Roitberg F, Ilbawi A. Delays and Disruptions in Cancer Health Care Due to COVID-19 Pandemic: Systematic Review. JCO Global Oncology. 2021;(7):311–23. doi: 10.1200/go.20.00639 . 33617304 PMC8081532

[8] Marron JM, Joffe S, Jagsi R, Spence RA, Hlubocky FJ. Ethics and resource scarcity: ASCO recommendations for the oncology community during the COVID-19 pandemic. J Clin Oncol. 2020;38(19):2201–5. doi: 10.1200/JCO.20.00960 32343643

[9] Knudsen KE, Willman C, Winn R. Optimizing the Use of Telemedicine in Oncology Care: Postpandemic Opportunities. Clinical Cancer Research. 2021;27(4):933–6. doi: 10.1158/1078-0432.CCR-20-3758 33229457 PMC7887011

[10] Koczwara B. Cancer survivorship care at the time of the COVID-19 pandemic. Med J Aust. 2020;213(3):107–8.e1. Epub 2020/06/28. doi: 10.5694/mja2.50684 . 32596806 PMC7361420

[11] Kenney LB, Vrooman LM, Lind ED, Brace-O’Neill J, Mulder JE, Nekhlyudov L, et al. Virtual visits as long-term follow-up care for childhood cancer survivors: Patient and provider satisfaction during the COVID-19 pandemic. Pediatr Blood Cancer. 2021:e28927–e. doi: 10.1002/pbc.28927 . 33559385 PMC7995169

[12] Moerdler S, Steinberg DM, Jin Z, Cole PD, Levy AS, Rosenthal SL. Well-Being of Pediatric Hematology Oncology Providers and Staff During the COVID-19 Pandemic in the New York and New Jersey Epicenter. JCO Oncology Practice. 2021;17(7):e925–e35. doi: 10.1200/OP.20.00882 . 33596094

[13] Yao KA, Attai D, Bleicher R, Kuchta K, Moran M, Boughey J, et al. Covid-19 related oncologist’s concerns about breast cancer treatment delays and physician well-being (the CROWN study). Breast Cancer Research and Treatment. 2021;186(3):625–35. doi: 10.1007/s10549-021-06101-1 33517522 PMC7847535

[14] Krok-Schoen JL, Pisegna JL, BrintzenhofeSzoc K, MacKenzie AR, Canin B, Plotkin E, et al. Experiences of healthcare providers of older adults with cancer during the COVID-19 pandemic. Journal of geriatric oncology. 2021;12(2):190–5. doi: 10.1016/j.jgo.2020.09.021 32978104 PMC7500913

[15] Penedo F, Cohen L, Bower J, Antoni M. COVID-19: Impact of the Pandemic and HRQOL in Cancer Patients and Survivors 2020 [cited 2021 October 15]. Available from: https://www.phenxtoolkit.org/toolkit_content/PDF/UMiami_HRQoL.pdf.

[16] Dillman DA, Bowker DK. The web questionnaire challenge to survey methodologists. Online social sciences. 2001:53–71.

[17] Dillman DA, Smyth JD, Christian LM. Internet, phone, mail, and mixed-mode surveys: the tailored design method: John Wiley & Sons; 2014.

[18] Harris PA, Taylor R, Thielke R, Payne J, Gonzalez N, Conde JG. Research electronic data capture (REDCap)—a metadata-driven methodology and workflow process for providing translational research informatics support. Journal of biomedical informatics. 2009;42(2):377–81. doi: 10.1016/j.jbi.2008.08.010 18929686 PMC2700030

[19] BrintzenhofeSzoc K, Krok-Schoen JI, Pisegna JL, MacKenzie AR, Canin B, Plotkin E, et al. Survey of cancer care providers’ attitude toward care for older adults with cancer during the COVID-19 pandemic. Journal of geriatric oncology. 2021;12(2):196–205. doi: 10.1016/j.jgo.2020.09.028 33144071 PMC7534786

[20] Sadler D, DeCara JM, Herrmann J, Arnold A, Ghosh AK, Abdel-Qadir H, et al. Perspectives on the COVID-19 pandemic impact on cardio-oncology: results from the COVID-19 International Collaborative Network survey. Cardio-oncology. 2020;6(1):1–13. doi: 10.1186/s40959-020-00085-5 33292763 PMC7691954

[21] Radhakrishnan VS, Nair RKS, Goel G, Ramanan V, Chandy M, Nair R. COVID-19 and haematology services in a cancer centre from a middle-income country: adapting service delivery, balancing the known and unknown during the pandemic. ecancermedicalscience. 2020;14. 10.3332/ecancer.2020.1110 PMC7581330 33144878

[22] Le Blanc PM, Hox JJ, Schaufeli WB, Taris TW, Peeters MC. Take care! The evaluation of a team-based burnout intervention program for oncology care providers. Journal of applied psychology. 2007;92(1):213. doi: 10.1037/0021-9010.92.1.213 17227163

[23] De la Fuente-Solana EI, Cañadas-De la Fuente GA, editors. Prevalence, risk factors, and levels of burnout among oncology nurses: A systematic review. Oncology Nursing Forum; 2016: Oncology Nursing Society. 10.1188/16.ONF.E104-E120 27105202

[24] Tetzlaff ED, Hylton HM, Ruth KJ, Hasse Z, Hall MJ. Changes in Burnout among oncology physician assistants between 2015 and 2019. JCO Oncology Practice. 2022;18(1):e47–e59. doi: 10.1200/OP.21.00051 34292762 PMC8757962

[25] Tetzlaff ED, Hylton HM, DeMora L, Ruth K, Wong Y-N. National Study of Burnout and Career Satisfaction Among Physician Assistants in Oncology: Implications for Team-Based Care. Journal of Oncology Practice. 2018;14(1):e11–e22. doi: 10.1200/JOP.2017.025544 . 29190209 PMC6528779

[26] Cañadas‐De la Fuente GA, Gómez‐Urquiza JL, Ortega‐Campos EM, Cañadas GR, Albendín‐García L, De la Fuente‐Solana EI. Prevalence of burnout syndrome in oncology nursing: a meta‐analytic study. Psycho‐oncology. 2018;27(5):1426–33. doi: 10.1002/pon.4632 29314432

[27] Pfefferbaum B, North CS. Mental health and the Covid-19 pandemic. New England Journal of Medicine. 2020;383(6):510–2. doi: 10.1056/NEJMp2008017 32283003

[28] Kumar A, Nayar KR. COVID 19 and its mental health consequences. Journal of Mental Health. 2020;180(6):817–8. doi: 10.1080/09638237.2020.1757052 32339041

[29] Galbraith N, Boyda D, McFeeters D, Hassan T. The mental health of doctors during the COVID-19 pandemic. BJPsych bulletin. 2020:1–4. 32340645 10.1192/bjb.2020.44 PMC7322151

[30] Werner EA, Aloisio CE, Butler AD, D’Antonio KM, Kenny JM, Mitchell A, et al., editors. Addressing mental health in patients and providers during the COVID-19 pandemic. Seminars in Perinatology; 2020: Elsevier. doi: 10.1016/j.semperi.2020.151279 32972778 PMC7373005

[31] Liu Q, Luo D, Haase JE, Guo Q, Wang XQ, Liu S, et al. The experiences of health-care providers during the COVID-19 crisis in China: a qualitative study. The Lancet Global Health. 2020;8(6):e790–e8. doi: 10.1016/S2214-109X(20)30204-7 32573443 PMC7190296

[32] Bansal P, Bingemann TA, Greenhawt M, Mosnaim G, Nanda A, Oppenheimer J, et al. Clinician wellness during the COVID-19 pandemic: extraordinary times and unusual challenges for the allergist/immunologist. The Journal of Allergy and Clinical Immunology: In Practice. 2020;8(6):1781–90. e3. doi: 10.1016/j.jaip.2020.04.001 32259628 PMC7129776

[33] Hlubocky FJ, Symington BE, McFarland DC, Gallagher CM, Dragnev KH, Burke JM, et al. Impact of the COVID-19 Pandemic on Oncologist Burnout, Emotional Well-Being, and Moral Distress: Considerations for the Cancer Organization’s Response for Readiness, Mitigation, and Resilience. JCO Oncology Practice. 0(0):OP.20.00937. doi: 10.1200/op.20.00937 33555934

[34] Thomaier L, Teoh D, Jewett P, Beckwith H, Parsons H, Yuan J, et al. Emotional health concerns of oncology physicians in the United States: Fallout during the COVID-19 pandemic. PLOS ONE. 2020;15(11):e0242767. doi: 10.1371/journal.pone.0242767 33232377 PMC7685431

[35] Lai J, Ma S, Wang Y, Cai Z, Hu J, Wei N, et al. Factors associated with mental health outcomes among health care workers exposed to coronavirus disease 2019. JAMA network open. 2020;3(3):e203976–e. doi: 10.1001/jamanetworkopen.2020.3976 32202646 PMC7090843

[36] Selvaraja VK, Gudipudib DK. Impact of the COVID-19 pandemic on work routine, practice and mental state of radiation oncologists in India: an online survey. ecancermedicalscience. 2021;15. doi: 10.3332/ecancer.2021.1165 33680079 PMC7929773

[37] Gill S, Hao D, Hirte H, Campbell A, Colwell B. Impact of COVID-19 on Canadian medical oncologists and cancer care: Canadian Association of Medical Oncologists survey report. Current Oncology. 2020;27(2):71. doi: 10.3747/co.27.6643 32489248 PMC7253737

[38] Chew QH, Wei KC, Vasoo S, Chua HC, Sim K. Narrative synthesis of psychological and coping responses towards emerging infectious disease outbreaks in the general population: practical considerations for the COVID-19 pandemic. Tropical Journal of Pharmaceutical Research. 2020;61(7). doi: 10.11622/smedj.2020046 32241071 PMC7926608

[39] Shanafelt TD, Boone S, Tan L, Dyrbye LN, Sotile W, Satele D, et al. Burnout and satisfaction with work-life balance among US physicians relative to the general US population. Archives of internal medicine. 2012;172(18):1377–85. doi: 10.1001/archinternmed.2012.3199 22911330

[40] Hlubocky FJ, Shanafelt TD, Back AL, Paice JA, Tetzlaff ED, Friese CR, et al. Creating a blueprint of well-being in oncology: an approach for addressing burnout from ASCO’s clinician well-being taskforce. American Society of Clinical Oncology Educational Book. 2021;41:e339–e53. doi: 10.1200/EDBK_320873 34061565

