# Supplementary material for: Oncology provider experiences during the COVID-19 pandemic
Source: PLoS One. 2022 Jul 26;17(7):e0270651. doi: 10.1371/journal.pone.0270651 (PMC9321423; doi:10.1371/journal.pone.0270651)
Supplement: S1 Table — (PDF) [file pone.0270651.s001.pdf]

Provider survey

Please complete the survey below.

Thank you!

Do you provide post-treatment cancer survivorship care to patients?	◯ Yes<br>◯ No
Do you live in a U.S. state or are you part of a U.S. tribe or territory?	◯ Yes<br>◯ No
Please note that responses deemed as fraudulent will NOT be eligible for an incentive	
Please write the word "sage" below.	______________________

You are invited to participate in a study under the direction of Mandi L. Pratt-Chapman, PhD (IRB #NCR202819). In order to participate, you must provide cancer survivorship care. You will be one of approximately 225 participants.

Before you decide to be a part of this study, you need to understand the risks and benefits.

Your decision to take part in the study is voluntary. You are free to choose whether or not you will take part in the study. You may stop completing the survey at any time.

PURPOSE
The purpose of this study is to understand the experiences of cancer care providers during COVID-19, also known as the coronavirus pandemic. We will ask you to complete a survey that should take approximately 5 minutes.

The study's results will give us information about how COVID-19 has affected cancer survivorship clinicians. We will compare provider experiences with survivor experiences during the pandemic.

POSSIBLE RISKS
There are no physical or research-related risks associated with this study. Possible discomforts include talking about your experiences with COVID-19.

In any study, there is a risk of loss of confidentiality. We will store your responses in a secure database only accessible to the research team. The records for this study will be kept private. In any published articles or presentations, we will not include any information that will make it possible to identify you as a subject. Your records for the study may be reviewed by departments of the University responsible for overseeing research safety compliance and financial audits.

COLLECTION OF PERSONAL INFORMATION
We will ask you information about your sociodemographic information to help us understand possible differences in the experiences of people who participate in this study.

We are required to collect certain personal information for tax purposes if you wish to receive the $50 incentive payment-including your name, address, zip code, and email address. You can participate in the study and not enter this information, but we cannot send you the $50 payment without this information.

POSSIBLE BENEFITS
There are no personal benefits to you in participating in this study, but your contribution may improve the quality of care provided to cancer survivors and the understanding of cancer survivorship care provider supports needed as a result of the COVID-19 pandemic.

COSTS
There are no monetary costs to you associated with this study.

QUESTIONS

If you have any questions about the procedures of this research study, please contact study PI Mandi L. Pratt-Chapman at mandi@gwu.edu. If you have any questions about the informed consent process or any other rights as a research study participant, please contact The George Washington University Office of Human Research at 202-994-2715.

Please check "Yes" if you wish to consent to participate in the survey. Please check "No" or close the browser if you do not wish to participate.

- ◯ Yes
- ◯ No

What is your professional role?

- ☐ Physician - Medical Oncologist
- ☐ Physician - Surgical Oncologist
- ☐ Physician - Radiation Oncologist
- ☐ Physician/Other
- ☐ Physician - Pathologist
- ☐ Nurse Practitioner
- ☐ Physician Assistant
- ☐ Nurse
- ☐ Dentist
- ☐ Clinical researcher
- ☐ Pharmacist
- ☐ Social worker
- ☐ Patient navigator
- ☐ Other

What is your professional role?

____

What type of health organization do you work at? (check all that apply)

- ☐ COC accredited
- ☐ NCI designated
- ☐ Academic/Teaching hospital
- ☐ Community hospital
- ☐ Free standing oncology practice
- ☐ Free standing multi-specialty group
- ☐ Other cancer practice
- ☐ Other

What type of healthcare organization do you work at?

____

Please indicate which CoC programs you are part of?

- ☐ Comprehensive Community Cancer Program
- ☐ Community Cancer Program
- ☐ Integrated Network Cancer Program
- ☐ Academic Comprehensive Cancer Program
- ☐ NCI-Designated Comprehensive Cancer Program
- ☐ Veterans Affairs Cancer Program
- ☐ Hospital Associate Cancer Program
- ☐ Pediatric Cancer Program
- ☐ NCI-designated Network Cancer Program
- ☐ Free Standing Cancer Center Program

Which of the following changes have occurred at your institution as a result of COVID-19? (check all that apply)

- ☐ Follow up for patients in treatment canceled or delayed
- ☐ Post-treatment survivorship care canceled or delayed
- ☐ Remote work for non-clinical staff
- ☐ Triage based on specialty guidelines (e.g. NCCN, ACOS, ASCO) to ensure clinical need for in-person visits
- ☐ Changes to treatment selection to reduce in-person visits (e.g., prioritization of oral over infusion chemotherapy when possible, selection of therapies with longer intervals, reduced length of adjuvant therapies, curative v. palliative intent of treatment plan)
- ☐ Team-based staff schedules to avoid potential cross-team exposure to COVID-19
- ☐ Suspension of nonurgent clinical trials
- ☐ Changes to visitor policies (e.g., restricting family from attending appointments with patients)
- ☐ Other

What changes not described in the previous question have occurred at your institution as a result of COVID-19?

____

Which of the following describe your experience? (check all that apply)

- ☐ I feel anxious about getting COVID-19
- ☐ I have been diagnosed with COVID-19
- ☐ I worry about possibly infecting others
- ☐ I am concerned about a family member or close friend getting or dying from COVID-19
- ☐ A family member or close friend has died from COVID-19
- ☐ I worry about the possibility of dying from COVID-19
- ☐ I feel I have no control over how COVID-19 will impact my life
- ☐ I believe there will be a vaccine or cure for COVID-19 soon
- ☐ I am hopeful that the COVID-19 pandemic will end soon
- ☐ None of these apply to me

In the last two weeks, please indicate which of the following is true for you? (check all that apply)

- ☐ I have experienced feelings of sadness or depression
- ☐ I feel negative and/or anxious about the future
- ☐ I have experienced changes in my sleep
- ☐ I have experienced changes in my eating and/or weight changes
- ☐ I have experienced difficulty concentrating
- ☐ I have experienced feelings of social isolation or loneliness
- ☐ I am hopeful about the future
- ☐ I make an effort to engage in mindfulness activities like meditation and/or yoga
- ☐ I have a hobby that brings joy to my life
- ☐ None of these apply to me

The next several questions ask about your thoughts on telehealth

Which of the following pre-treatment visits do you think are appropriate for telehealth? (check all that apply)

- ☐ New oncology appointment or consultation (e.g., diagnosis, prognosis, care plan)
- ☐ Discussion of goals of care
- ☐ Discussions about imaging or lab results
- ☐ Chemotherapy education
- ☐ Genetics counseling
- ☐ Plastic surgery education visit
- ☐ Multi-disciplinary consultation
- ☐ Clinical trial recruitment
- ☐ Second opinion
- ☐ None of the above
- ☐ Other

What other pre-treatment visits are appropriate for telehealth?

____

Which of these follow-up visits do you think are appropriate for telehealth? (check all that apply)

- ☐ Surgical wound evaluation (with video)
- ☐ Assessment of a symptom or medical concern related to cancer and treatment (e.g., nausea, fatigue)
- ☐ Management of a symptoms related to cancer and treatment (e.g. side effects from treatment)
- ☐ Comorbidity management (non-cancer conditions)
- ☐ Clinical trial visit
- ☐ None of the above
- ☐ Other

What other follow-up visits do you think are appropriate for telehealth?

____

Which of the following supportive care services do you think are appropriate for telehealth? (check all that apply)

- ☐ Nutrition consultation
- ☐ Financial counseling
- ☐ Tobacco/ smoking cessation
- ☐ Occupational, physical or speech therapy
- ☐ Lymphedema care
- ☐ Plastic surgery
- ☐ Patient navigation support
- ☐ Social work or mental health visit
- ☐ Support groups
- ☐ Fertility visit
- ☐ Sexual health visit
- ☐ Traditional Healer visit
- ☐ Spiritual Care visit
- ☐ Palliative (pain relief) care visit
- ☐ End-of-life care visit
- ☐ None of the above
- ☐ Other

What supportive care services not mentioned in the previous questions do you think are appropriate for telehealth?

____

Have you seen any patients using telehealth (e.g. Zoom, Facetime, video or phone call)?

- ◯ Yes
- ◯ No

Approximately how many telehealth visits have you had with patients?	◯ 1-25 visits<br>◯ 25-50 visits<br>◯ 50-100 visits<br>◯ 100+ visits
Including any time troubleshooting technology barriers with the patient, was the length of your telehealth appointments...	◯ Shorter than typical in-person appointments<br>◯ Longer than typical in-person appointments<br>◯ About the same length of time as typical in-person appointments
What technical challenges did you experience during telehealth appointments? (check all that apply)	☐ Patient not familiar with technology<br>☐ Patient failing to login for appointment<br>☐ Screen freezing<br>☐ Audio challenges<br>☐ Other<br>☐ None
What other technical challenges did you experience during telehealth appointments?	__________________________________
What benefits did you experience in using telehealth? (check all that apply)	☐ Protecting patients from infection risk<br>☐ Protecting healthcare workers from infection risk<br>☐ Ability to observe patient's home situation<br>☐ Ability to include family members who live in other locations in the telehealth visit<br>☐ Improved efficiency of appointments<br>☐ Other
What benefits not previously listed did you experience with telehealth?	__________________________________
Thinking about your use of telehealth with patients over the last 6 months, please indicate challenges you experienced. (check all that apply)	☐ Limited training on using telehealth<br>☐ Issues with multi-jurisdictional licensure<br>☐ Concerns about complying with legal standards<br>☐ Difficulty capturing reimbursement for services<br>☐ Worry about the privacy of my patients' information when using telehealth<br>☐ Worry about missing something important for the patient (e.g. unable to do physical exam)<br>☐ Unable to meet patient needs adequately<br>☐ Did not always have interpretation services when needed<br>☐ Worry about litigation when using telehealth<br>☐ Other<br>☐ None
What other challenges to telehealth did you experience?	__________________________________

Thinking about your use of telehealth with patients over the last 6 months, please indicate which of the following describe your perspective. (check all that apply)	☐ I could easily talk to the patient using telehealth<br>☐ I could hear the patient clearly using telehealth<br>☐ I was able to express myself effectively<br>☐ I could see the patient as well as if we met in person<br>☐ I feel comfortable communicating with patients using telehealth<br>☐ Telehealth is an acceptable way to delivery healthcare services<br>☐ I would offer telehealth services again<br>☐ Other
What else describes your perspective?	__________________________________
What long-term benefits do you think will result from COVID-19 healthcare changes? (check all that apply)	☐ Improved provider comfort with telehealth<br>☐ Improved technologic capacity for telehealth<br>☐ Improved protocols for future pandemics<br>☐ Regular use of telehealth<br>☐ Improved risk triage protocols for patient care<br>☐ Improved visit efficiency (e.g. intake paperwork sent to patients in advance of appointment)<br>☐ Improved timeliness and access for patients<br>☐ Other
What other long-term benefits do you think will result from COVID-19 healthcare changes	__________________________________

How concerned are you about the following issues after physical distancing protocols for coronavirus are lifted?

	Very concerned	Somewhat concerned	Not concerned
Inability for system to handle backlogged patient needs	○	○	○
Progression of cancer in patients who experienced care delays	○	○	○
Progression of cancer in patients afraid of accessing in-person care	○	○	○

Please describe any other concerns related to cancer survivorship care after physical distancing protocols for coronavirus are lifted? ____

Please provide any recommendations to improve cancer services based on your experiences during the COVID-19 pandemic ____

We are now going to ask some questions about your demographics

What is your sex assigned at birth?

- ◯ Male
- ◯ Female
- ◯ Intersex
- ◯ I prefer not to answer

What is your gender identity (check all that apply)

- ☐ Cisgender male (sex assigned at birth and gender are the same)
- ☐ Transgender male (sex assigned at birth and gender are different)
- ☐ Cisgender female (sex assigned at birth and gender are the same)
- ☐ Transgender female (sex assigned at birth and gender are different)
- ☐ Gender fluid / Gender non-conforming
- ☐ Two Spirit
- ☐ Other
- ☐ I prefer not to answer
- ☐ I do not understand the question

What is your sexual orientation (check all that apply)

- ☐ Gay/ Lesbian/ Homosexual
- ☐ Bisexual/ Pansexual
- ☐ Straight/ Heterosexual
- ☐ Queer
- ☐ Two spirit
- ☐ Other
- ☐ I prefer not to answer
- ☐ I do not understand the question

What is your race/ethnicity (check all that apply)

- ☐ Asian
- ☐ Black
- ☐ Hispanic / Latinx
- ☐ Native American / Alaska Native/ Pacific Islander
- ☐ White
- ☐ Other
- ☐ I prefer not to answer

How would you describe your race/ethnicity?

____

How would you describe the location where you practice?

- ◯ Urban (city)
- ◯ Suburban (outside a city)
- ◯ Rural (not near a city)

Where do you primarily practice?

- ◯ State
- ◯ Tribe
- ◯ Territory

What state or territory do you practice in?

- ☐ Alabama
- ☐ Alaska
- ☐ Arizona
- ☐ Arkansas
- ☐ California
- ☐ Colorado
- ☐ Connecticut
- ☐ Delaware
- ☐ Florida
- ☐ Georgia
- ☐ Hawaii
- ☐ Idaho
- ☐ Illinois
- ☐ Indiana
- ☐ Iowa
- ☐ Kansas
- ☐ Kentucky
- ☐ Louisiana
- ☐ Maine
- ☐ Maryland
- ☐ Massachusetts
- ☐ Michigan
- ☐ Minnesota
- ☐ Mississippi
- ☐ Missouri
- ☐ Montana
- ☐ Nebraska
- ☐ Nevada
- ☐ New Hampshire
- ☐ New Jersey
- ☐ New Mexico
- ☐ New York
- ☐ North Carolina
- ☐ North Dakota
- ☐ Ohio
- ☐ Oklahoma
- ☐ Oregon
- ☐ Pennsylvania
- ☐ Rhode Island
- ☐ South Carolina
- ☐ South Dakota
- ☐ Tennessee
- ☐ Texas
- ☐ Utah
- ☐ Vermont
- ☐ Virginia
- ☐ Washington
- ☐ West Virginia
- ☐ Wisconsin
- ☐ Wyoming
- ☐ District of Columbia

What territory or tribe do you practice in?

____

Forte

In order to recieve payment for your participation please complete the below

The first 225 participants to complete the survey will be offered a $50 payment. If you would like to forgo this payment to encourage more cancer providers to complete the survey and further the knowledge we can gather from this survey, you can select "no" on receiving your payment. If you choose to receive the payment, please select "yes" to continue on to enter information for payment.	◯ Yes<br>◯ No

The study team apologizes in advance that its accounting system is not LGBTQI friendly. The IRS requires that we collect this information to ensure that research participants who receive more than $600 incentives in any one year pay the appropriate taxes. Please complete this information as you would on your tax forms. If you do not wish to complete the form, you do not have to, but we will be unable to send you your $50 payment. Thank you for understanding these constraints.

First Name	__________________________
Middle Name	__________________________
Last name	__________________________
Suffix	__________________________
Email	__________________________
Address	________________________________
Date of birth (MM/DD/YYYY)	__________________________
Gender	◯ Male<br>◯ Female
